# A Visible Colorimetric Fluorescent Probe for Hydrogen Sulfide Detection in Wine

**DOI:** 10.1155/2019/2173671

**Published:** 2019-01-10

**Authors:** Haitao Chen, Xiaoming Wu, Shaoxiang Yang, Hongyu Tian, Yongguo Liu, Baoguo Sun

**Affiliations:** Beijing Advanced Innovation Center for Food Nutrition and Human Health, Beijing Key Laboratory of Flavor Chemistry, Beijing Technology and Business University, No. 11 Fucheng Road, Haidian District, Beijing 100048, China

## Abstract

A new efficient and practical fluorescent probe 6-(benzo[d]thiazol-2-yl)naphthalen-2-yl-thiophene-2-carboxylate (probe **1**) was synthesized to detect hydrogen sulfide (H_2_S). The addition of H_2_S caused the solution of probe **1** to change from colorless to yellow, and the solution of probe **1** changes to different colors with respect to different concentrations of H_2_S. Importantly, probe **1** could help detect H_2_S efficiently by a distinct color response as a visible detection agent. Probe **1** reacted with various concentrations of H_2_S (0–200 *μ*M), and the detection limit for H_2_S was 0.10 *μ*M. Particularly, probe **1** can be applied as a sensor to detect H_2_S accurately in wine samples.

## 1. Introduction

Hydrogen sulfide (H_2_S) has unpleasant rotten egg smell [1, 2]. H_2_S is a significant compound in wine, and a detection threshold value is measured from 1.1 to 1.6 *μ*g/L [3]. Alcoholic fermentation is mainly a way to generate H_2_S because of enzymatic catabolism of S-amino acid and yeast from elemental sulfite pesticide residues, sulfate, or sulfur [4]. Due to abiotic storage of wine, the level of hydrogen sulfide keeps an increasing trend. Other sources of H_2_S are investigated all the time [5, 6]. H_2_S affects the quality of wines and causes economic losses [7, 8].

H_2_S is an important part in the processes of physiological and pathophysiological responses, and abnormal levels of H_2_S cause various diseases [9, 10], including cardiac ischemia disease [11], hypertension [12], atherosclerosis [13], diabetes [14], tumor [15], and other diseases. Therefore, the sensitive and selective methods for detecting H_2_S in wine are required.

The methods to detect H_2_S include colorimetry [16], electrochemical precipitation [17], metal-induced sulfide precipitation [18], gas chromatography [19], high-performance liquid chromatography-mass spectrometry [6], and sulfide precipitation [20]. Recently, fluorescent probes have been considered a practical tool for H_2_S detection [21–24]. The H_2_S fluorescent probes are designed by some approaches, such as sulfide-induced precipitation of quantum dots [25, 26], reduction of azide and nitro group to amines [27–32], substitution reaction [33], nucleophilic reactions [34, 35], high adsorption of S^2−^ to Cu^2+^ [36], and the reaction with the unsaturated double bond [37]. Recently, different kinds of fluorescent probes have been designed and compounded to detect H_2_S in living cells, and development of efficient and practical sensors to detect H_2_S in wine is still crucial [38]. In order to discover a more responsive and visible colorimetric fluorescent probe, a new fluorescent probe (probe **1**) was introduced in this work, with naphthalene and benzothiazole ring moiety as the fluorophore and thiophene-2-carboxylate as the reaction site. Probe **1** shows responsive and visible colorimetric precipitation for H_2_S with naked eye. Especially, the solution of probe **1** poses different colors at different H_2_S concentrations under ambient light. Probe **1** could be used as a sensor to obtain H_2_S levels and to obtain high recovery in real wine samples.

## 2. Materials and Methods

### 2.1. General Methods

The chemicals and reagents were purchased from Beijing Huaxue Shiji (Beijing, P.R. China). The reagents were all analytically pure. ^1^H-NMR and ^13^C-NMR spectra were recorded at 400 MHz and 100 MHz, respectively. Chemical shifts (*δ*) were expressed in ppm relative to TMS, and coupling constants (*J*) are in Hz. The high-resolution mass spectrum (HRMS) was performed at a Bruker Apex IV FTMS. Fluorescence spectra were recorded on a Hitachi F-4600 fluorescence spectrometer with a temperature controller.

### 2.2. Preparation of Probe **1**

**Scheme 1:**
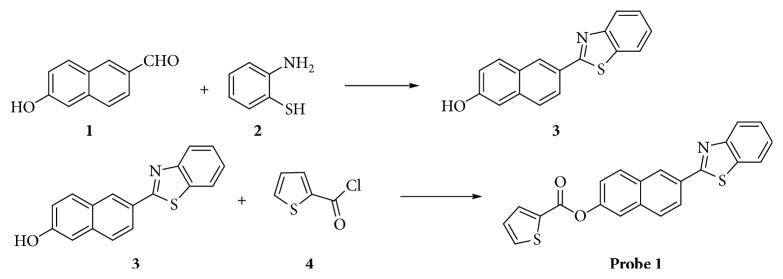
Synthesis of probe **1**.

*6-(benzo[d]thiazol-2-yl)naphthalen-2-ol*. 6-hydroxy-2-naphthaldehyde (0.86 g, 5 mmol) and 2-aminothiophenol (0.63 g, 5 mmol) were dissolved in ethanol (25 mL) and stirred for 15 min. And then, p-toluenesulfonic acid (0.34 g, 2 mmol) in ethanol (5 mL) was added into the mixture slowly. The reaction mixture was heated in an oil bath at 80°C overnight. After the mixture was cooled to room temperature, the mixture was added 50 mL distilled water. The precipitate was collected by evaporation and dried to yield 6-(benzo[d]thiazol-2-yl)naphthalen-2-ol as a yellow solid (1.29 g, 93.5%, Scheme 1).

^1^H NMR (300 MHz, DMSO), *δ* (ppm): 10.11 (s, 1H), 8.54 (s, 1H), 8.06 (m, 4H), 7.84 (d, *J* = 8.67 Hz, 1H), 7.53 (td, *J* = 7.29, 1.17 Hz, 1H), 7.43 (td, *J* = 7.95, 1.17 Hz, 1H), and 7.16 (m, 2H). ^13^C NMR (75 MHz, DMSO): *δ* (ppm): 168, 158, 154, 137, 135, 131, 128, 127, 126, 125, 123, 120, and 109.

Probe **1**: *6-(benzo[d]thiazol-2-yl)naphthalen-2-yl-thiophene-2-carboxylate*. 6-(benzo[d]thiazol-2-yl)naphthalen-2-ol (0.50 g, 1.8 mmol), CHCl_3_ (20 mL), and thiophene-2-carbonyl chloride (0.29 g, 1.8 mmol) was added. After stirring 20 min, a drop of trimethylamine was dissolved in CHCl_3_ (5 mL) and added slowly. The reaction mixture was heated at 80°C for 4 h. The precipitate used evaporation to be collected and then used column chromatography to purify probe **1** as a solid (0.570 g, 81.8%; Scheme 1).

^1^H NMR (300 MHz, DMSO), *δ* (ppm): 8.78 (s, 1H), 8.28 (d, *J* = 4.35 Hz, 2H), 8.20 (d, *J* = 3.96 Hz, 1H), 8.12–8.14 (m, 4H), 8.11 (d, *J* = Hz, 1H), 7.96 (s, 1H), 7.58 (t, *J* = 4.41 Hz, 2H), 7.50 (t, *J* = 3.78 Hz, 1H), and 7.35 (s, 1H). ^13^C NMR (75 MHz, DMSO): *δ* (ppm): 168, 161, 154, 150, 136, 135, 132, 131, 130, 129, 128, 127, 126, 125, 123, and 120. HRMS: calcd. [M]^+^ 387.0388; 387.0390.

### 2.3. Preparation of Solutions of Probe **1** and Analytes

The HPLC-grade DMSO as reagent was used to dissolved probe **1**. After mixing, probe **1** stock solution was obtained. Analytes NaF, Na_2_SO_3_, NaCl, NaHSO_3_, NaNO_3_, NaBr, Na_2_SO_4_, Na_2_S_2_O_3_, Na_2_S_2_O_5_, NaS_2_O_6_, CH_3_COONa, NaHCO_3_, and Na_2_S used distilled water to be dissolved and obtained 10 mM aqueous solutions. Various concentrations could be obtained by using distilled water to dilute the stock solutions.

### 2.4. Preparation of Wine Samples

Three kinds of beers and four kinds of red wines were bought from wumart supermarket (Beijing) and different concentrations of Na_2_S were added (Na_2_S is the H_2_S source), and the 504 nm fluorescence signals of samples were recorded.

### 2.5. The Procedures of H_2_S Determination and Samples Analysis

The ready of the detection system: dimethyl sulfoxide (0.48 mL) and probe solution (0.02 mL) were mixed. And then buffer solution was added and made up to 2 mL in the cuvette. After mixing, the spectrum was tested by recording the fluorescence signals.

Fluorescence spectrophotometer parameters: excitation wavelength, 330 nm; emission wavelength, 504 nm; temperature, 37°C; voltage, 700 v; slit width, 5 nm/5 nm.

## 3. Results and Discussion

### 3.1. Fluorescent Probe Preparation

Probe **1** was synthesized in just a two-step reaction. First, the intermediate compound **3** was manufactured by nucleophilic addition reaction and cyclodehydration of compound **1** with compound **2**. Second, probe **1** was obtained so that compound **3** and thiophene-2-carbonyl chloride (compound **4**) performed an esterification reaction. This synthetic process and the purification of silica gel column chromatographic separation were easy. ^1^H NMR and ^13^C NMR (Figures S1 and S2) were used to determine the structure of 6-(benzo[d]thiazol-2-yl)naphthalene-2-ol, light yellow powder. The structure of Probe **1** was characterized by ^1^H NMR, ^13^C NMR, and HRMS (Figures S3–S5).

### 3.2. Sensing Property of Probe **1** towards H_2_S

**Figure 1:**
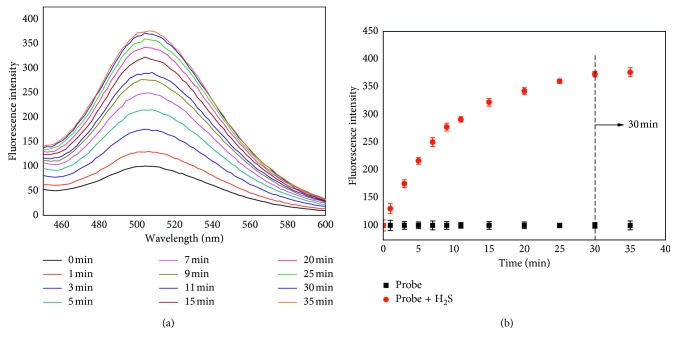
(a) Time-dependent fluorescence spectra of probe **1** (10 *μ*M) in the presence of H_2_S (200 *μ*M) in phosphate buffer saline (PBS; pH 7.4) with DMSO (v/v, 3 : 1) at 37°C; (b) time-dependent fluorescence intensity changes of probe **1** (10 *μ*M) in the presence of H_2_S (200 *μ*M) at 504 nm. *λ*_ex_ = 307 nm, *λ*_em_ = 504 nm, and slit width = 5 nm/5 nm. The test was repeated 3 times.

The fluorescence response of probe **1** (10 *μ*M) to Na_2_S (we used Na_2_S for H_2_S production) was firstly verified in 10 mM phosphate buffer saline (PBS; pH 7.4) in DMSO at 37°C. According to Figure 1(a), the fluorescence intensity was detected at 1, 3, 5, 7, 9, 11, 15, 20, 25, 30, and 35 min after 200 *μ*M H_2_S was added, and the fluorescence intensity increased almost three times. The fluorescence signal at 504 nm increased all the time until 30 min (Figure 1(b)). The results suggest that probe **1** shows good response to H_2_S in neutral environment.

**Figure 2:**
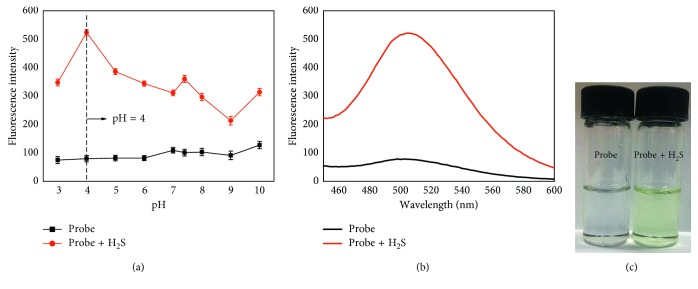
(a) Fluorescent intensity of probe **1** (10 *μ*M) in the absence and presence of H_2_S (200 *μ*M) in different pH buffer solutions with DMSO (v/v, 3 : 1). The test was repeated 3 times; (b) fluorescence spectra of probe **1** (10 *μ*M) and probe **1** (10 *μ*M) with H_2_S (200 *μ*M) in buffer solution (pH 4.0) with DMSO (v/v, 3 : 1) at 37°C; (c) the color change of probe **1** (10 *μ*M) in the absence and presence of H_2_S (200 *μ*M).

The fluorescent response of probe **1** to H_2_S in different pH values (Table S1) from 3.0 to 10.0 was investigated (Figure 2(a)). The data suggest that the fluorescent intensity of probe **1** did not change in various pH values. However, as H_2_S was added, the fluorescent intensity of probe **1** increased quickly from 3.0 to 4.0 and decreased from 4.0 to 9.0. The fluorescence intensity showed largest differences between probe **1** and probe **1**-H_2_S in pH 4.0. The water solubility of H_2_S was reported as an equilibrium between molecular and ionic forms (H_2_S ⇌ HS^−^ ⇌ S^2−^) [39]. The pKa values for the first and second dissociation steps are 7.0 and 12.0, respectively [39]. The major form of hydrogen sulfide exists as HS^−^ with a minor form of free H_2_S in pH 7.4 [39] and exists as free H_2_S in pH 4.0. The fluorescent intensity of probe **1-**H_2_S decreased indicating that the reaction activity of probe **1** to H_2_S decreased. So, the reaction activity of probe **1** to H_2_S decreased from 4.0 to 7.0, increased from 4.0 to 7.4, and then decreased from 7.4 to 9.0. As probe **1** can identify H_2_S, HS^−^, and S^2−^ [40], the reaction activity of probe **1** to H_2_S with pH is generally not too obvious regularity. The above results reveal that the pH value of 4.0 is better suitable for further studies.

The fluorescence response of probe **1** (10 *μ*M) to H_2_S was verified in 10 mM buffer solution (pH 4.0) in DMSO at 37°C, the fluorescence signal at 504 nm increases all the time until 30 min (Figure S6). The fluorescence response of free probe **1** (10 *μ*M) and H_2_S (200 *μ*M) added to probe **1** in buffer solution (pH 4.0) is shown in Figure 2(b). Meanwhile, the solution color changed from colorless to yellow (Figure 2(c)). All results indicate that probe **1** was a turn-on fluorescent probe and could be applied to detect H_2_S in this experimental condition by the naked eye.

**Figure 3:**
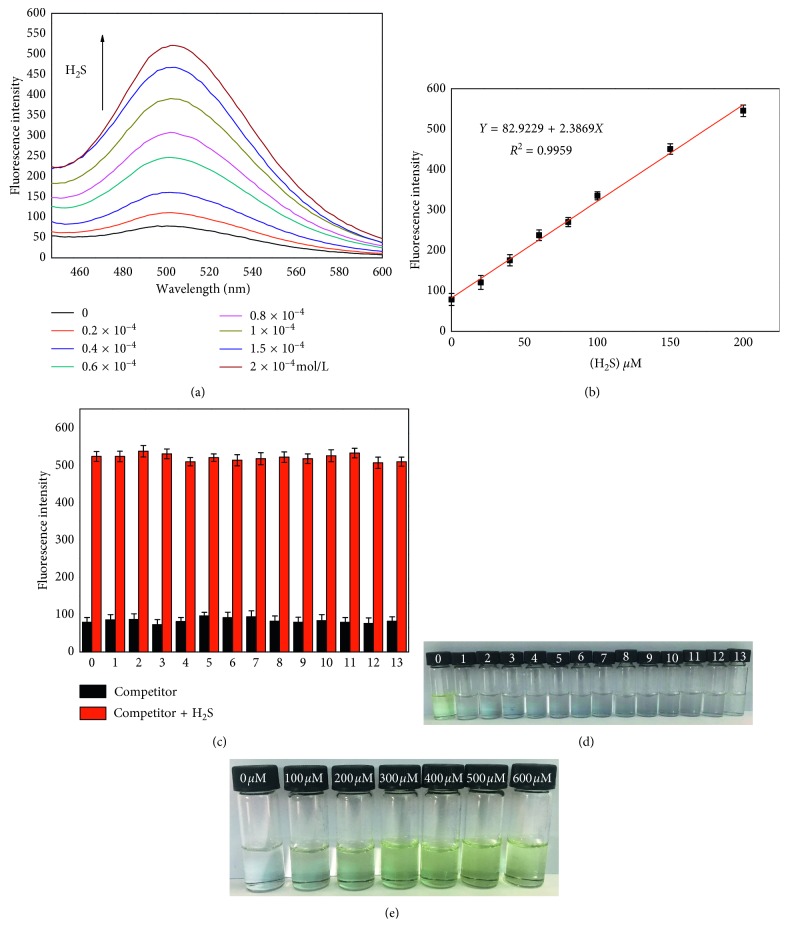
(a) Fluorescence spectra of probe **1** (10 *μ*M) with H_2_S (0–200 *μ*M); (b) the plot of fluorescence intensity difference with H_2_S from 0 to 200 *μ*M in buffer solution (10 mM, pH 4.0) with DMSO (v/v, 3 : 1); (c) fluorescence intensity change of probe **1** (10 *μ*M) upon addition of various species (200 *μ*M for each. 0, blank; 1, GSH; 2, F^−^; 3, Cl^−^; 4, Br^−^; 5, SO_3_^2−^; 6, HSO_3_^−^; 7, S_2_O_3_^2−^; 8, S_2_O_5_^2−^; 9, S_2_O_6_^−^; 10, CH_3_COO^−^; 11, SO_4_^2−^; 12, HCO_3_^−^; 13, CO_3_^2−^. 200 *μ*M for H_2_S). Wavelength, 504 nm. The test was repeated 3 times; (d) the solution color of probe 1 with Na_2_S and competing species (200 *μ*M for each. 0, H_2_S; 1, GSH; 2, F^−^; 3, Cl^−^; 4, Br^−^; 5, SO_3_^2−^; 6, HSO_3_^−^; 7, S_2_O_3_^2−^; 8, S_2_O_5_^2−^; 9, S_2_O_6_^−^; 10, CH_3_COO^−^; 11, SO_4_^2−^; 12, HCO_3_^−^; 13, CO_3_^2−^. 200 *μ*M for H_2_S); (e) photograph of probe **1** (10 *μ*M) at different H_2_S concentrations under ambient light in buffer solution (pH 4.0) with DMSO (v/v, 3 : 1) at 25°C.

The solution of probe **1** in buffer solution (pH 4.0) was added with different concentrations of H_2_S (0–200 *μ*M), and the change of fluorescence intensity was recorded. As shown in Figure 3(a), a highest fluorescence peak was shown at 504 nm, and the fluorescence intensity was increasing with the addition of H_2_S. The highest of fluorescence intensity was reached in the presence of 200 *μ*M H_2_S. The data could make a good linearity, *R* ^2^ = 0.9959 (Figure 3(b)). The detection limit (LOD) of probe **1** for H_2_S was 0.1 *μ*M, based on *C*_im_ = 3 SD/B according to the definition from IUPAC. These results suggest that pH 4.0 was the best pH value for probe **1** to detect H_2_S and provide a nice quantitative detection method for H_2_S.

To verify the selectivity of probe **1** for H_2_S, GSH, F^−^, Cl^−^, Br^−^, SO_3_^2−^, HSO_3_^−^, S_2_O_3_^2−^, S_2_O_5_^2−^, S_2_O_6_^−^, CH_3_COO^−^, SO_4_^2−^, HCO_3_^−^, and CO_3_^2−^ in buffer solution (pH 4.0) was chosen as the complex condition to research the fluorescent response of probe **1**. As shown in Figure 3(c), under this condition, the competitor did not cause the fluorescence change obviously. At the same time, competition experiments were conducted by adding H_2_S to the probe **1** solutions containing the above analytes. Fluorescent response of probe **1** shows that fluorescence had no changes toward H_2_S and H_2_S + competitor. It clearly indicated that the presence of competitor did not interfere with H_2_S detection. In addition, only H_2_S caused the probe **1** solution to change color from colorless to yellow (Figure 3(d)). Based on the above result, it indicated that probe **1** has good recognition toward H_2_S in complex environment. Using probe **1** solution develops a test strip system (10 *μ*M; buffer solution: DMSO = 3 : 1, pH 4.0). The test strip system showed different color changes to different concentrations of H_2_S ranging from 0 *μ*M to 600 *μ*M (Figure 3(e)). So, these data show that probe **1** can be used to develop an easy-to-detect test strip system for an effective method to monitor H_2_S by the naked eye.

### 3.3. Reaction Mechanism

**Scheme 2:**
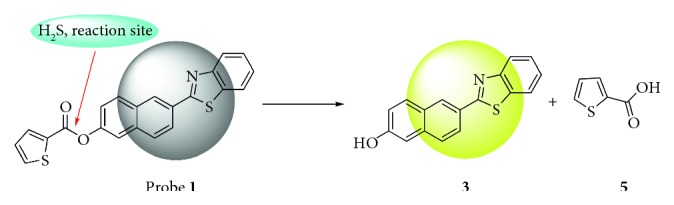
The mechanism for probe **1** with H_2_S.

A possible response mechanism may attribute to H_2_S-induced hydrolysis of thenoic acid ether moiety in probe **1** and thereby generate 6-(benzo[d]thiazol-2-yl)naphthalen-2-ol (compound **3**) and 2-thiophenecarboxylic acid (compound **5**), as shown in Scheme 2. To verify the response mechanism mentioned above, reaction of probe **1** with H_2_S was analyzed by GC-MS (Figure S6). A peak at 5.40 min, *m*/*z* = 142.0 was the reaction product generated by the esterification reaction of compound **6** with methanol. Peak at 22.18 min, *m*/*z* = 277.1, which correlated to the formation of compound **3**. The results suggest that probe **1** can react with H_2_S efficiently and verify the proposed mechanism.

### 3.4. Detection of H_2_S in Wine

As H_2_S negatively affects wine quality, it is an important reason to cause faulty wine. The data of probe **1** to detect H_2_S in real samples were recorded to prove the actual practicability of probe **1**. Three kinds of beers and four kinds of red wines were bought from Wumart supermarket (Beijing) and were added to the solution of probe **1** (10 *μ*M; pH 4.0). Then, H_2_S of different concentration levels (50 *μ*M and 100 *μ*M) were added. The fluorescence intensity of all these samples was investigated at 504 nm.

As shown in Table 1, 0.53, 0.69, 0.74, and 0.49 *μ*M were obtained in four red wine samples. 0.41, 0.28, and 0.32 *μ*M were founded in three beer samples. Probe **1** can detect H_2_S concentration in red wine and beer, and the recovery ranged from 90.65% to 110.00% showing that probe **1** has good practicability to detect H_2_S levels in real samples. The results show that the probe **1** as a testing method is feasible and practical to determinate H_2_S in wine.

Probe **1** is compared with some previously reported H_2_S fluorescent probe in terms of detection range, detection limit, and practical applications as listed in Table S2. Majority of H_2_S fluorescent probes have been designed and used for biological imaging, but H_2_S fluorescent probes for wine are rare. In this work, probe **1** has different color changes for different concentrations of H_2_S ranging from 0 *μ*M to 600 *μ*M. Probe **1** has a wider detection range (0–200 *μ*M) than our previous fluorescent probes and reported H_2_S fluorescent probes (Table S2). Furthermore, probe **1** has successfully been used to detect H_2_S concentrations in red wine and beer. In addition, the visual change indicates that probe **1** can be used to develop a naked eye detection agent to detect H_2_S levels.

## 4. Conclusions

In summary, we developed a sensitive and visible colorimetric fluorescent probe to detect H_2_S. The function of probe **1** relies on H_2_S-induced make thenoic acid ether group cleave, and the produced fluorophores (6-(benzo[d]thiazol-2-yl)naphthalen-2-ol and compound **3** were verified by GC-MS studies. When probe **1** reacted with H_2_S, the solution color changed from colorless to yellow, and addition of different concentrations of H_2_S posed different color changes, indicating that probe **1** could be employed as a testing tool for H_2_S. Furthermore, our work shows that probe **1** has been successfully applied to test H_2_S levels in red wine and beer samples.

## Figures and Tables

**Table 1:** Determination of H_2_S concentrations in wine.

Sample	H_2_S level found (*µ*mol)	Added (*µ*mol)	H_2_S level found (*µ*mol)	Recovery (%)	*s* ^2^
Red wine A	0.53	50	53.02	104.88	0.007
		100	100.21	99.68	0.005
Red wine B	0.69	50	48.90	96.47	0.001
		100	98.14	97.33	0.004
Red wine C	0.74	50	46.10	90.65	0.005
		100	97.14	96.28	0.005
Red wine D	0.49	50	47.96	94.99	0.004
		100	98.12	97.64	0.006
Beer A	0.41	50	50.71	100.59	0.004
		100	100.32	99.91	0.005
Beer B	0.28	50	49.10	97.65	0.002
		100	100.34	100.00	0.006
Beer C	0.32	50	55.36	110.00	0.001
		100	105.43	105.09	0.005

The test was repeated 3 times.

## Data Availability

The data used to support the findings of this study are included within the article.

## Supplementary Materials

Supplementary Materials Experiment section. Figure S1: ^1^H NMR spectra of 6-(benzo[d]thiazol-2-yl)naphthalen-2-ol. Figure S2: ^13^C NMR spectra of 6-(benzo[d]thiazol-2-yl)naphthalen-2-ol. Figure S3: ^1^H NMR spectra of probe **1**. Figure S4: ^13^C NMR spectra of probe **1**. Figure S5: HRMS spectra of probe **1**. Figure S6: the time-dependent fluorescence spectra in pH 4.0 buffer. Figure S7: GC-MS spectra of probe **1**-H_2_S. Table S1: disposition of different pH buffer solutions. Table S2: comparison of fluorescent probes for H_2_S.
