# Supplementary material for: A Visible Colorimetric Fluorescent Probe for Hydrogen Sulfide Detection in Wine
Source: J Anal Methods Chem. 2019 Jan 10;2019:2173671. doi: 10.1155/2019/2173671 (PMC6348852; doi:10.1155/2019/2173671)
Supplement: Supplementary Materials — Experiment section. Figure S1: 1H NMR spectra of 6-(benzo[d]thiazol-2-yl)naphthalen-2-ol. Figure S2: 13C NMR spectra of 6-(benzo[d]thiazol-2-yl)naphthalen-2-ol. Figure S3: 1H NMR spectra of probe 1. Figure S4: 13C NMR spectra of probe 1. Figure S5: HRMS spectra of probe 1. Figure S6: the time-dependent fluorescence spectra in pH 4.0 buffer. Figure S7: GC-MS spectra of probe 1-H2S. Table S1: disposition of different pH buffer solutions. Table S2: comparison of fluorescent probes for H2S. [file 2173671.f1.doc]

**A visible colorimetric fluorescent probe for hydrogen sulfide detection in wine**

Haitao Chen, Xiaoming Wu, Shaoxiang Yang*****, Hongyu Tian, Yongguo Liu, Baoguo Sun

*Beijing Advanced Innovation Center for Food Nutrition and Human Health, Beijing Key laboratory of Flavor Chemistry, Beijing Technology and Business University, No.11 Fucheng Road, Haidian District, Beijing 100048,* *People’s Republic of China*

^*^ Telephone: +86-10-68985382. Fax: 86-10-68985382. E-mail:

[yangshaoxiang@th.btbu.edu.cn](mailto:yangshaoxiang@th.btbu.edu.cn) (Shaoxiang Yang)

| **TABLE OF CONTENTS** | **PAGE** |
| --- | --- |
| **Experiment section** | **2** |
| **Figure S1. ^1^H NMR spectra of 6-(benzo[d]thiazol-2-yl)naphthalen-2-ol** | **3** |
| **Figure S2. ^13^C NMR spectra of 6-(benzo[d]thiazol-2-yl)naphthalen-2-ol** | **3** |
| **Figure S3. ^1^H NMR spectra of probe 1** | **4** |
| **Figure S4. ^13^C NMR spectra of probe 1** | **4** |
| **Figure S5. HRMS spectra of probe 1** | **5** |
| **Figure S6. The time-dependent fluorescence spectra in pH 4.0 buffer** | **6** |
| **Figure S7. GC-MS spectra of probe 1-H_2_S** | **7** |
| **Table S1**. **Disposition of different pH buffer solutions** | **8** |
| **Table S2**. **Comparison of fluorescent probes for H_2_S** | **8** |

**Experiment section**

The procedures of samples analysis

Dimethyl sulfoxide (0.48 mL) and probe solution (0.02 mL) were mixed. And then added buffer solution (pH 4.0) to made up to 2 mL in the cuvette. The red wine or beer (20 μL) were added to the cuvette. Then mixing, the spectrum was tested by recording the fluorescence signals.

Fluorescence spectrophotometer parameters, excitation wavelength: 330 nm; emission wavelength: 504 nm; temperature: 37 ^o^C; [v](http://dict.youdao.com/w/voltage/#keyfrom=E2Ctranslation)oltage: 700 v; slit width: 5 nm, 5 nm.

**Figure S1. ^1^H NMR spectra of 6-(benzo[d]thiazol-2-yl)naphthalen-2-ol**

**Figure S2. ^13^C NMR spectra of 6-(benzo[d]thiazol-2-yl)naphthalen-2-ol**

**Figure S3. ^1^H NMR spectra of probe 1**

**Figure S4. ^13^C NMR spectra of probe 1**

**Figure S5. HRMS spectra of probe 1**

**
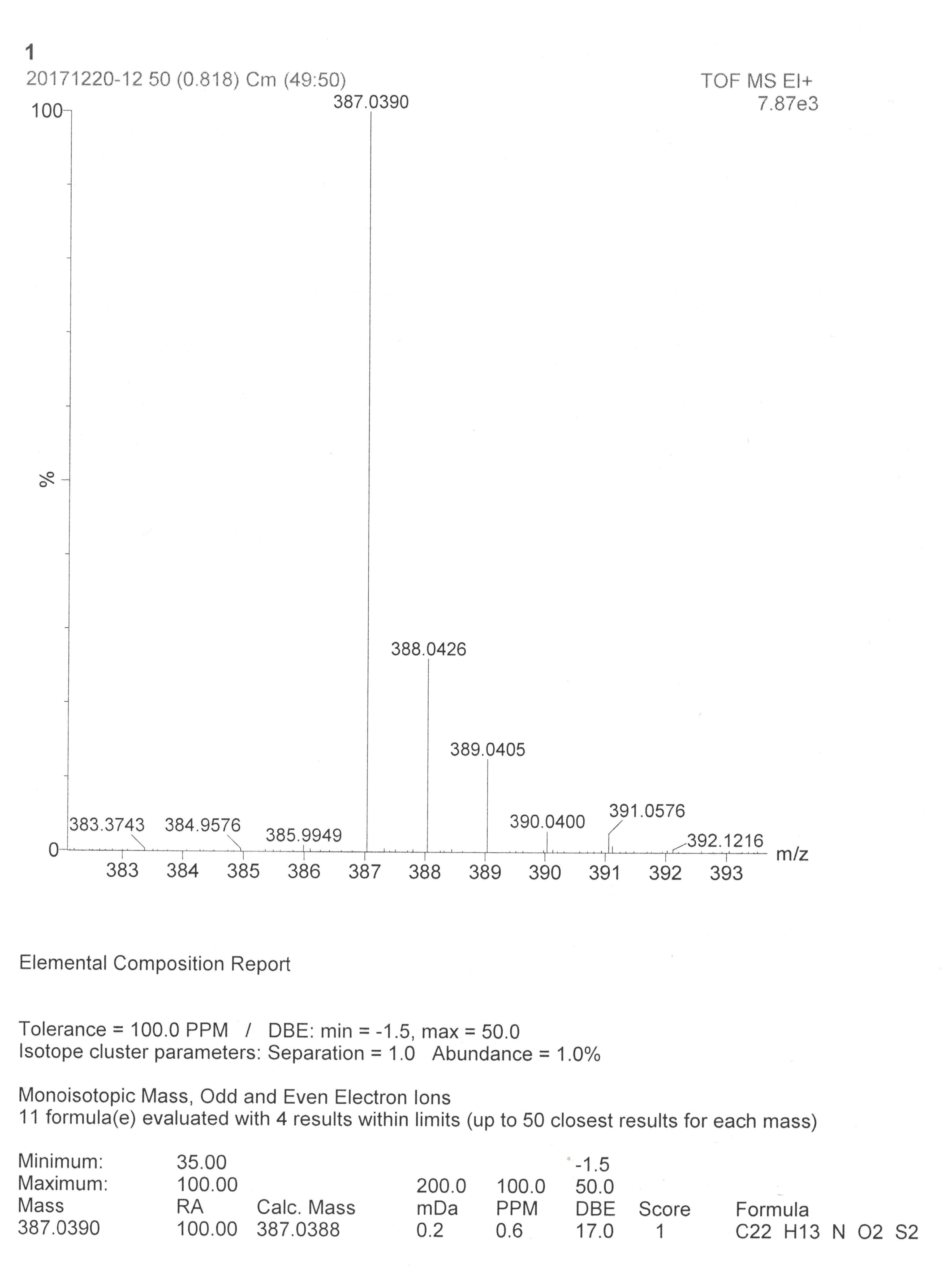
**

**Figure S6. The time-dependent fluorescence spectra in pH 4.0 buffer**


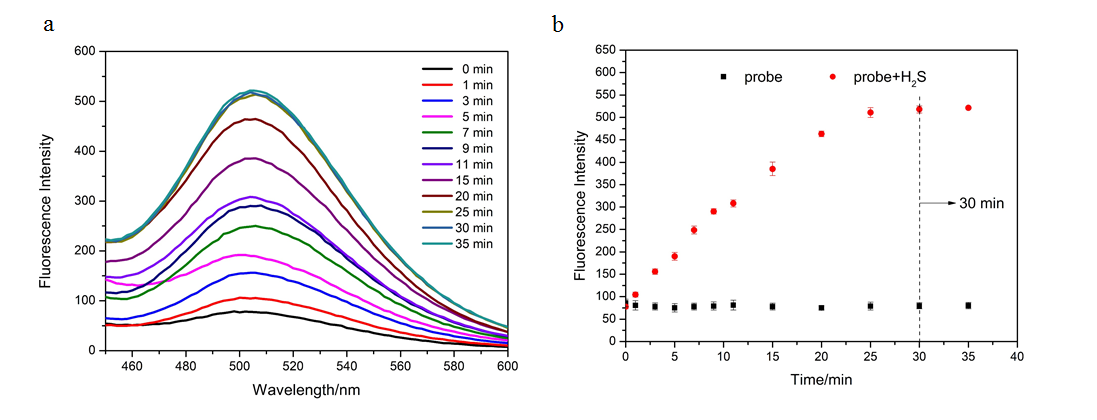


(a) Time-dependent fluorescence spectra of probe 1 (10 μM) in the presence of H_2_S (200 μM) in buffer (pH 4.0) with DMSO (v/v, 3:1) at 37 °C; (b) Time-dependent fluorescence intensity changes of probe **1** (10 μM) in the presence of H_2_S (200 μM) at 504 nm. *λ*_ex_=307 nm, *λ*_em_=504 nm, slites: 5 nm/5 nm. The test was repeated 3 times.

**Figure S7. GC-MS spectra of probe 1-H_2_S**


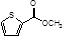


**Table S1. Disposition of different pH buffer solutions**

| pH | A/mL | B/mL |
| --- | --- | --- |
|  | Na_2_HPO_4·_12H_2_O (0.2mol/L) | citric acid (0.1mol/L) |
| 3 | 4.11 mL | 15.89 mL |
| 4 | 7.71 mL | 12.29 mL |
| 5 | 10.30 mL | 9.70 mL |
|  | NaH_2_PO_4·_2H_2_O (0.2mol/L) | Na_2_HPO_4·_12H_2_O (0.2mol/L) |
| 6 | 87.7 mL | 12.3 mL |
| 7 | 39 mL | 61 mL |
| 7.4 | 19 mL | 81 mL |
| 8 | 5.3 mL | 94.7 mL |
| 9 | Na_2_CO_3_ (0.60g) + NaH CO_3_ (3.7g)+ H_2_O (100 mL) | |
| 10 | NaHCO_3_ (50mL, 0.05mol/L) + NaOH (10.7 mL, 0.1mol/L) + H_2_O (39.3 mL) | |

**Table S2.** **Comparison of fluorescent probes for H_2_S**

| Entry | Fluorescent probes | Detection limit | Detection  range | Response time | Applications | References |
| --- | --- | --- | --- | --- | --- | --- |
| 1 |  | 42 nM | 10-100μM | 6 min | living cells imaging | *Chem. Commun.* **2016**, *52*, 4628−4631 |
| 2 |  | 3.2 μM | 0-50μM | 5 min | living cells imaging | *Chem. Commun.* **2016**, *52*, 7016−7019 |
| 3 |  | 24 nM | 0-20μM | 60 min | living cells imaging | *Angew.Chem. Int. Edit.* **2016**, *55*, 9993–9996 |
| 4 |  | 2.46 μM | 0-100μM | 40 min | living cells imaging | *Anal. Chem.* **2016**, *88*, 5476–5481 |
| 5 |  | 0.1 μM | 0-8μM | 250 s | living cells imaging | *Anal. Chem.* **2016**, *88*, 7206–7212 |
| 6 |  | 1 μM | 20-100μM | 15 min | living cells imaging | *Anal. Chem.* **2016**, *88*, 11253–11260 |
| 7 |  | 0.7 μM | 0-100μM | 20 min | living cells imaging | *Anal. Chem.* **2016**, *88*, 11892–11899 |
| 8 |  | 0.22 μM | 0-80μM | 4 min | living cells imaging | *Anal. Methods.* **2016**, 8, 8022–8027 |
| 9 |  | 90 nM | 0-36μM | 24 min | living cells imaging | [*Sens. Actuators B.* **2016**, *232*, 705–711](https://www.ncbi.nlm.nih.gov/pmc/articles/PMC4853739/) |
| 10 |  | 0.10 μM | 0-30μM | 150 s | living cells imaging | [*Sens. Actuators B.* **2016**, *235*, 691–697](https://www.ncbi.nlm.nih.gov/pmc/articles/PMC4853739/) |
| 11 |  | 26 nM | 0-60μM | 3 min | living cells imaging | *Spectrochim. Acta. A*. **2016**, *168*, 132–138 |
| 12 |  | 37 nM | 0-20μM | 5 min | living cells imaging | *Anal.Methods,***2017***, 9, 2859*–2864 |
| 13 |  | 41 nM | 0-20μM | 20 min | living cells imaging | *Chem. Commun.* **2017**, *53*, 4791−4794 |
| 14 |  | 1.65 μM | 0-70μM | 6 min | living cells imaging | [*Sens. Actuators B.* **2017**, *248*, 50–56](https://www.ncbi.nlm.nih.gov/pmc/articles/PMC4853739/) |
| 15 |  | 150 nM | 0-50μM | 500 s | living cells imaging | *Sens. Actuators B.* **2017**, *238*, 619–625 |
| 16 |  | 50 nM | 0-20μM | 20 min | living cells imaging | *Anal. Chem.* **2017**, *89*, 4587–4594 |
| 17 |  | 2.5 nM | 0-15μM | 20 min | living cells imaging | *Anal. Chem.* **2017**, *89*, 1801–1807 |
| 18 |  | 5.2 nM | 0-30μM | 10 min | living cells imaging | *New J. Chem.* **2017**, *41*, 3367–3373 |
| 19 |  | 108 nM | 0-60μM | 45 min | living cells imaging | *Dyes and Pigments* **2017***,138,*112–118 |
| 20 |  | 48 nM | 0-16μM | 30 min | living cells imaging | *Dyes and Pigments* **2017***, 139,*482–486 |
| 21 |  | 71 nM | 0-80μM | 25 min | living cells imaging | [*RSC Adv.*](http://dx.doi.org/10.1039/2046-2069/2011) **2017**, ***7***, 15817–15822 |
| 22 |  | 83 nM | 0-5μM | 30 min | cellular imaging | *Biosens. Bioelectron.* **2017**, 91, 699–705 |
| 23 |  | 56 nM | 0-40μM | 10 min | red wine | *Chin. J. Chem.* **2017**, 35, 477—482 |
| 24 |  | 1.27 μM | 0-50μM | 3 h | cellular imaging | *Talanta* **2018***,* 181, 104—111 |
| 25 |  | 0.33 μM | 0-100μM | 10 min | cellular imaging | *Sens. Actuators B.* **2018**, *260*, 264–273 |
| 26 |  | 5.7 nM | 0-20μM | 30 min | cellular imaging | *Org. Biomol. Chem.* **2018***, DOI:* 10.1039/C7OB02641B |
| 27 |  | 30 nM | 0-25μM | 38 min | red wine; beer | *J. Food. Sci.* **2018***,* 83, 108–112 |
| 28 |  | 18 nM | 0-20 μM | 15 min | red wine; beer | *Food Anal. Method.* **2018***, 11,* 1398−1404 |
| 29 |  | 0.10 μM | 0-200 μM | 30 min | red wine; beer | ***This work*** |
